# Characteristics, risk factors and outcomes of gastrointestinal hemorrhage in COVID-19 patients: A meta-analysis

**DOI:** 10.12669/pjms.37.5.4351

**Published:** 2021

**Authors:** Jie Chen, Ying Hang

**Affiliations:** 1 Jie Chen Dept. of General Surgery, Shanghai Jiaotong University Affiliated Sixth People’s Hospital, Shanghai, China; 2 Ying Hang Dept. of Emergency, Renji Hospital, School of Medicine, Shanghai Jiaotong University, Shanghai, China

**Keywords:** Bleeding, Coronavirus, Hemorrhage, Hospital admission, Mortality

## Abstract

**Background:**

The severe acute respiratory syndrome coronavirus 2 (SARS-CoV-2), a cause of coronavirus disease 2019 (COVID-19), mainly targets the respiratory system. However, recent studies also show its role in causing gastrointestinal hemorrhage, potentially affecting morbidity and mortality-related outcomes of the patients. There is still no consensus on the risk factors, characteristics, and the overall outcome of the gastrointestinal hemorrhage in COVID-19 patients. The main aim of this study was to summarize current evidence, assessing risk factors that promote the onset of gastrointestinal hemorrhage in COVID-19 patients, and to compare the incidences of the different sites of gastrointestinal lesions, the events of abdominal pain, diarrhea, intensive care unit admissions, and mortality between COVID-19 patients with or without gastrointestinal bleeding.

**Methods:**

A search of the academic literature was performed according to the PRISMA guidelines across five databases i.e., Web of Science, EMBASE, CENTRAL, Scopus, and MEDLINE. A random-effect meta-analysis was conducted to analyze the influence of the history of drugs consumption, gastrointestinal bleeding, the different incidence of gastrointestinal lesions, events of abdominal pain, intensive care unit (ICU) admission, and mortality between COVID-19 patients with/without gastrointestinal bleeding.

**Results:**

Out of 458 studies, three eligible studies with 663 participants (mean age: 69.7 ± 4.3 years) were included. A meta-analysis showed a medium-to-large influence of the history of gastrointestinal bleeding (Hedge’s g: 1.01) and anticoagulant drug consumption (g: 0.33) on the gastrointestinal bleeding in COVID-19 patients. Moreover, the incidence of gastroduodenal ulcers was higher as compared to esophagitis (37.5% versus 9.9%).

**Conclusions:**

The study provides preliminary evidence regarding the risk factors associated with the onset of gastrointestinal hemorrhage among COVID-19 patients. The study also outlines the characteristics and the outcomes of gastrointestinal hemorrhage in COVID-19 patients.

## INTRODUCTION

Novel acute respiratory coronavirus disease (COVID-19) is a widely transmissible respiratory viral disease that has led to a worldwide pandemic affecting almost 96,093,690 people worldwide.^1^ COVID-19 has been primarily reported to have pulmonological manifestations. However, recent evidence has demonstrated that COVID-19 can also aggravate deficits in the mucosal barrier of the gastrointestinal tract.^2^–^4^ Recent observational and epidemiological studies have reported the onset of hemorrhage in the upper and lower gastrointestinal tract in as many as 2% to 13% of patients with COVID-19.^5^,^6^ These patients exhibit a wide array of symptomatic manifestations including abdominal pain, nausea, diarrhea, and vomiting^3^ that may complicate treatment, eventually leading to poorer morbidity- and mortality-related outcomes.^7^,^8^

Typically, the management of COVID-19 related symptoms is done by the administration of non-steroidal anti-inflammatory drugs that can inhibit the marked inflammatory response of the host system, a major cause of pulmonary damage and mortality. Prophylactics using anticoagulants are also recommended to prevent micro- and macro-thromboembolic risks posed by COVID-19.^9^ Although the administration of anticoagulant and non-steroidal anti-inflammatory drugs is beneficial in improving short- and long-term morbidity and mortality outcomes in COVID-19 patients,^7^,^10^ their combined administration is strongly associated with increased events of gastrointestinal hemorrhage.^11^ In case of COVID-19, there is little evidence of a possible effect of these drugs on the risk of gastrointestinal hemorrhage.

To date, only few individual case control studies have attempted to evaluate the incidence of different types of gastrointestinal lesions in COVID-19 patients^12^–^14^ with conflicting results. While one study has reported a moderate incidence of esophagitis (28.9%) (Gonzalez et al.)^12^, others have reported a low incidence, ranging between 3 to 5% (13,14). These contradicting results, together with the lack of meta-analysis data and consensus on the risk factors, characteristics, and outcomes of gastrointestinal hemorrhage in COVID-19 patients, present challenges for clinicians.

Therefore, the main goal of this current meta-analysis was to gain a better understanding of the overall impact of gastrointestinal hemorrhage in COVID-19 patients by evaluating its risk factors (history of drug consumptions, gastrointestinal bleeding), characteristics (events of abdominal pain, diarrhea, type of gastrointestinal lesions), and the outcomes (intensive care unit admission events, mortality).

## METHODS

Meta-analysis was performed based on the PRISMA (Preferred Reporting Items for Systematic Reviews and Meta-Analyses) guidelines.^15^

### Data search strategy

The literature search was performed in five scientific databases (Web of science, MEDLINE, CENTRAL, EMBASE, and Scopus) from inception till December 2020, across a combination of MeSH keywords, including “Gastrointestinal bleeding”, “COVID-19”, “coronavirus”, “comorbidity”, and “mortality”. The bibliography section of the included studies was manually searched to identify further relevant studies. The inclusion criteria were as follows:

- Studies, evaluating the influence of the history of drug consumption, gastrointestinal bleeding on the outcome of gastrointestinal bleeding in COVID-19 patients.
- Studies, evaluating clinical manifestations, types of ulcers, and outcomes (ICU admission, mortality) in COVID-19 patients with gastrointestinal bleeding.
- Studies of human participants.
- Case control studies, prospective trials or retrospective cohort trials.
- Studies published in peer-reviewed scientific journals.
- Studies published in English.

The screening of the studies was independently performed by two reviewers.

### Data analysis

A within-group meta-analysis was performed using CMA, Comprehensive Meta-analysis version 2.0^16^ based on the random-effects model.^17^ Weighted effect size, Hedge’s g, was calculated to determine the history of drug consumption (non-steroidal anti-inflammatory drugs, anticoagulants), history of gastrointestinal bleeding, events of abdominal pain and diarrhea between groups with/without gastrointestinal bleeding in COVID-19 patients. The event rate of gastrointestinal lesions (gastroduodenal ulcer, esophagitis), as well as rate ratios for intensive care unit admission and overall mortality outcomes between groups with/without gastrointestinal bleeding in COVID-19 patients were evaluated. The heterogeneity among the studies was assessed by I^2^ statistics. I^2^ statistics between 0-25% was considered as negligible heterogeneity, 25%-75% moderate heterogeneity and ≥75% substantial heterogeneity.^18^ Publication bias was evaluated by Duval and Tweedy’s trim and fill procedure^19^ that imputates the studies from either side of the plotted graph to identify any unbiased effect. The significance level for this study was determined at 5%.

### Ethical approval:

Not applicable.

## RESULTS

**Fig.1:**
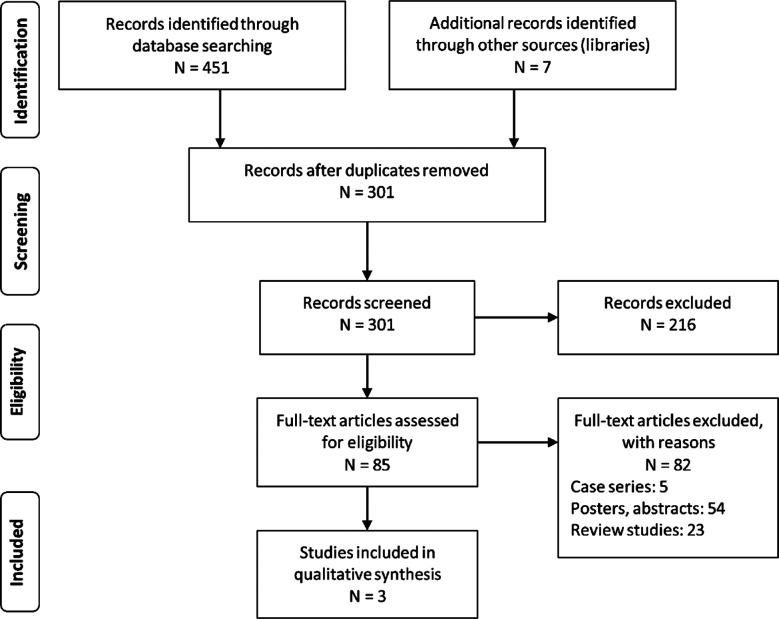
PRISMA flowchart.

A search across academic databases provided a total of 451 studies. Additional seven studies were identified during the screening of the reference sections of the included studies. A total of three studies met inclusion criteria (Fig.1). All of the included studies were retrospective cohort studies.^12^–^14^

### Participant information

Selected studies reported data from a total of 663 (287F, 376M) patients. Of them, 228 (96F, 132M) patients with COVID-19 also suffered from gastrointestinal bleeding, whereas 435 (191F, 244M) patients with COVID-19 did not exhibit gastrointestinal bleeding symptoms.

The average age of the participants was 69.79 ± 4.3 years, with an average age of 73.1 ± 4.4 years in the group of COVID-19 patients with gastrointestinal bleeding, and 67.61 ± 3.6 years in the group of COVID-19 patients without gastrointestinal bleeding.

### Publication bias

**Fig.2:**
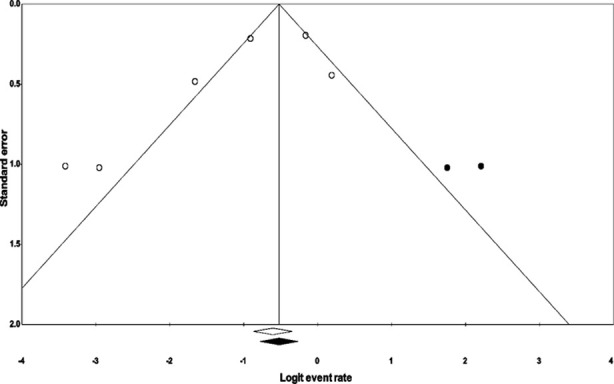
Publication bias. Publication bias of the included studies, evaluated by Duval & Tweedy’s trim and fill method.

We used Duval and Tweedy’s trim and fill method to determine missing studies according to the random effect model on either side of the mean effect of the funnel plot. The method identified that two studies were missing on the right side of the standard mean effect. Based on the random effect models, the standard point estimate and the 95% intervals were determined as 0.25 (0.13 to 0.42) overall for all the studies. Based on the trim and fill imputation, the estimates were computed as 0.35 (0.20 to 0.54) (Fig.2).

### Meta-analysis report:

### History of non-steroidal anti-inflammatory drug consumption

**Fig.3:**
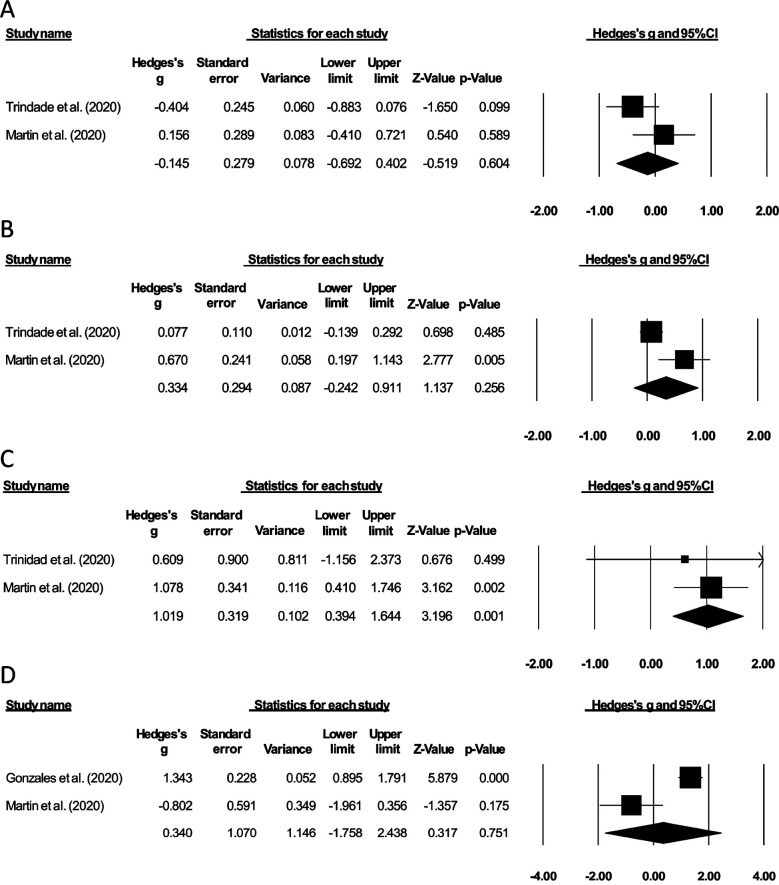
Forest plots of the included studies: comparison of COVID-19 patients with and without gastrointestinal bleeding. (A) History of non-steroidal anti-inflammatory drug consumption, (B) History of anticoagulant consumption, (C) History of gastrointestinal bleeding events, (D) Abdominal pain events. The effect sizes are presented as black boxes; 95% confidence intervals are presented as whiskers. Negative effect size: higher incidence of the evaluated parameter in the group of COVID-19 patients without gastrointestinal bleeding. Positive effect size: higher incidence of the evaluated parameter in the group of COVID-19 patients with gastrointestinal bleeding.

The history of non-steroidal anti-inflammatory drug consumption was evaluated by two studies.^12^,^14^ We observed a *small* negative effect size suggesting a lesser history of non-steroidal anti-inflammatory drug consumption for COVID-19 patients with gastrointestinal bleeding as compared to COVID-19 patients without gastrointestinal bleeding (Hedge’s g: -0.14, 95% C.I: -0.69 to 0.40, p=0.60), with no heterogeneity (I^2^: 0%) (Fig.3A).

### History of anticoagulant consumption

The history of anticoagulant consumption was evaluated in two studies^12^,^14^ with a *medium* positive effect size, suggesting a higher history of anticoagulant consumption for COVID-19 patients with gastrointestinal bleeding as compared to COVID-19 patients without gastrointestinal bleeding (Hedge’s g: 0.33, 95% C.I: -0.24 to 0.91, p=0.25), with no heterogeneity (I^2^: 0%) (Fig.3B).

### History of gastrointestinal bleeding

The history of gastrointestinal bleeding events was evaluated in two studies.^12^,^14^ A *large* positive effect size suggested g a higher history of gastrointestinal bleeding for COVID-19 patients with gastrointestinal bleeding as compared to COVID-19 patients without gastrointestinal bleeding (Hedge’s g: 1.01, 95% C.I: 0.39 to 1.64, p<0.01), with no heterogeneity (I^2^: 0%) (Fig.3C).

### Abdominal pain

The incidence of abdominal pain events was evaluated by two studies.^12^,^13^ We observed a *medium* positive effect size suggesting higher occurrence of abdominal pain for COVID-19 patients with gastrointestinal bleeding as compared to COVID-19 patients without gastrointestinal bleeding (Hedge’s g: 0.34, 95% C.I: -1.75 to 2.43, p=0.75), with no heterogeneity (I^2^: 0%) (Fig.3D).

### Diarrhea

**Fig.4:**
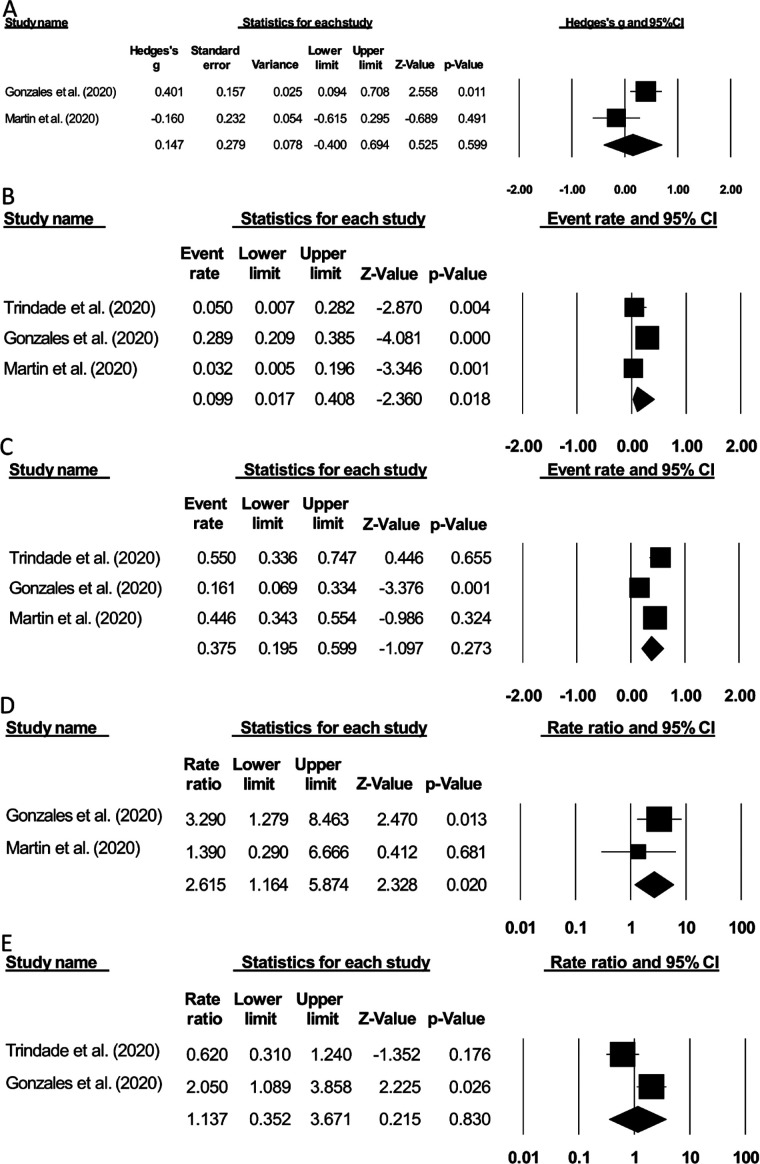
Forest plot of the included studies: comparison of COVID-19 patients with and without gastrointestinal bleeding. (A) Diarrhea events, (B) Esophagitis, (C) Gastroduodenal ulcer, (D) Intensive care unit admission, (E) Mortality. The effect sizes are presented as black boxes. 95% confidence intervals are presented as whiskers. Negative effect size: higher incidence of the evaluated parameters in the group of COVID-19 patients without gastrointestinal bleeding. Positive effect size: higher incidence of the evaluated parameter in the group of COVID-19 patients with gastrointestinal bleeding.

The incidence of diarrhea events was evaluated by two studies^12^,^13^ with a *small* positive effect size suggesting higher incidence of diarrhea for COVID-19 patients with gastrointestinal bleeding as compared to COVID-19 patients without gastrointestinal bleeding (Hedge’s g: 0.14, 95% C.I: -0.40 to 0.69, p=0.59), with no heterogeneity (I^2^: 0%) (Fig.4A).

### Esophagitis outcome

The incidence of esophagitis was evaluated in three studies.^12^–^14^ We observed a 9.9% event rate of esophagitis in COVID-19 patients with gastrointestinal bleeding (95% C.I: 1.7% to 40.8%, p=0.01), with no heterogeneity (I^2^: 0%) (Fig.4B).

### Gastroduodenal ulcer outcome

The incidence of the gastroduodenal ulcer was evaluated in three studies.^12^–^14^ We observed an event rate of 37.5% of gastroduodenal ulcer in COVID-19 patients with gastrointestinal bleeding (95% C.I: 19.5% to 59.9%, p=0.27), with minor heterogeneity (I^2^: 24.6%) (Fig.4C).

### Intensive care unit admission

The incidence of intensive care unit admission was reported by two studies. 12,13 Observed rate ratio of (2.61, 95% C.I: 1.16 to 5.87, p=0.02) (Fig.4D) suggested higher intensive care unit admissions for gastrointestinal bleeding cases with COVID-19, with no heterogeneity (I^2^: 0%).

### Mortality

The incidence of mortality was evaluated by two studies^12^,^14^ with a rate ratio of (1.13, 95% C.I: 0.35 to 3.67, p=0.83), suggesting higher mortality for gastrointestinal bleeding cases with COVID-19, with no heterogeneity (I^2^: 0%) (Fig.4E).

## DISCUSSION

This systematic review and meta-analysis provide for the first-time comprehensive evidence regarding the risk factors (history or NSAIDs and anticoagulant consumption), characteristics, and outcomes of gastrointestinal hemorrhage in COVID-19 patients. We showed higher incidence of prior anticoagulants consumption and gastrointestinal bleeding in COVID-19 patients with gastrointestinal hemorrhage. Moreover, we found that COVID-19 patients with gastrointestinal hemorrhage had higher incidences of abdominal pain, and diarrhea, higher rates of admissions to intensive care units, and higher mortality as compared to COVID-19 patients without gastrointestinal hemorrhage.

Widespread incidence of gastrointestinal hemorrhage in COVID-19 patients poses a challenge for clinicians worldwide.^20^ Studies show that the onset of systemic gastrointestinal manifestations can complicate the treatment course and worsen morbidity- and mortality-related outcomes for COVID-19 patients.^21^,^22^ Most importantly, gastrointestinal hemorrhage may impact administration of non-steroidal anti-inflammatory drugs^23^, and anticoagulants^24^ that may potentially counter pronounced inflammatory response and micro-, and macro-thromboembolic events associated with COVID-19. Recent studies have also suggested that prior long-term consumption of these drugs could potentially predispose COVID-19 patients to gastrointestinal complications. In our meta-analysis, we found that anticoagulants but not non-steroidal anti-inflammatory drugs were more frequently consumed by COVID-19 patients with gastrointestinal hemorrhage (Hedge’s g: 0.33 and -0.14 respectively), as compared to COVID-19 patients without gastrointestinal hemorrhage. Our findings are in line with previous reports, suggesting the detrimental influence of anticoagulant consumption on gastrointestinal hemorrhage.^25^–^27^ We also found that prior history of gastrointestinal bleeding among patients with COVID-19 was another strong predictor for the onset of gastrointestinal hemorrhage (g: 1.01).

We found a lack of consensus among the included studies as to the prevalence of the sites of gastrointestinal lesions. Gonzalez et al.^12^ reported a high prevalence of esophagitis (28.9%), whereas Trindade et al.^14^ and Martin et al.^13^ reported a low prevalence of esophagitis (3 to 5%) among COVID-19 patients. Similarly, Trindade et al.^14^, and Martin et al.^13^ reported a high prevalence of gastroduodenal ulcers (44.6% to 55%), while Gonzalez et al.^12^ reported a low prevalence of only 16.1% among COVID-19 patients. Our meta-analysis confirms the findings of the latter and reports a higher overall incidence rate of gastroduodenal ulcer (Event rate: 37.5%), as compared to esophagitis (EE: 9.9%) in COVID-19 patients.

Additionally, we attempted to classify the common symptomatic manifestations of gastrointestinal hemorrhage in COVID-19 patients. Martin et al.^13^ sub-classified hemorrhage symptoms in the upper and lower gastrointestinal tract and reported that while melena was the most common manifestation in the upper gastrointestinal tract, hematochezia was most common in the lower gastrointestinal tract. In our meta-analysis, two of the most common symptoms exhibited by COVID-19 patients with gastrointestinal hemorrhage were abdominal pain (g: 0.34) and diarrhea (g: 0.14). We attempted to develop a consensus regarding the outcomes of the gastrointestinal bleeding (intensive care admission and mortality) in COVID-19 patients. The evidence reported by the studies, included in the review, was inconclusive. Gonzalez et al.^12^ reported that the COVID-19 patients with gastrointestinal hemorrhage had higher odds (Odd’s ratio: 2.98) of intensive care unit admission as compared to COVID-19 patients without gastrointestinal hemorrhage. On the other hand, Martin et al.^13^ reported the opposite observation, with lesser odds (Odd’s ratio: 2.98) of intensive care unit admission events in COVID-19 patients with gastrointestinal hemorrhage. In our meta-analysis we found a significant influence (Rate ratio: 2.61, p=0.02) of gastrointestinal hemorrhage on ICU admission rates in COVID-19 patients. Moreover, the onset of gastrointestinal hemorrhage in COVID-19 patients was associated with increased mortality risk (RR: 1.13, p=0.83), although this effect was not statistically significant.

### Limitations of the study:

Despite being a novel study, the present systematic review and meta-analysis has several limitations. This study was not pre-registered in a systematic review repository such as PROSPERO York. We understand that the lack of prior registration could raise concerns for the validity of this current review.^28^ Additionally, our evaluation of the risk factors, characteristics, and outcomes could be biased due to insufficient data. For example, two out of the three included studies in our meta-analysis reported the outcome of a history of drug use (i.e. non-steroidal anti-inflammatory drugs, anticoagulants) among 331 patients.^13^,^14^ Similarly, two studies had reported the clinical manifestations (abdominal pain, diarrhea) among 455 patients.^12^,^13^ Although these parameters did not show heterogeneity in our analysis, we strongly recommend our readers to interpret these results with caution. Future case control studies are needed to evaluate the outcomes of risk factors, characteristics and outcomes associated with gastrointestinal hemorrhage in COVID-19 patients, and to share descriptive data in open access data repositories. The evaluation of these outcomes would be highly beneficial for clinicians to determine best practice guidelines to manage gastrointestinal hemorrhage in patients with COVID-19.

## CONCLUSION

We provide preliminary evidence of risk factors, characteristics, and outcomes associated with gastrointestinal hemorrhage in COVID-19 patients. We show that prior history of anticoagulants consumption and gastrointestinal bleeding are prominent risk factors for the development of gastrointestinal hemorrhage in COVID-19 patients. We report a higher incidence of gastroduodenal ulcer as compared to esophagitis as the bleeding source, and higher incidence of ICU admissions and mortality in COVID-19 patients with gastrointestinal hemorrhage. These findings can be used in developing best practice guidelines for the management of gastrointestinal hemorrhage in patients with COVID-19.

### Authors’ contributions:

**JC** conceived and designed the study.

**JC and YH** collected the data and performed the literature search.

**JC** was involved in the writing of the manuscript.

All authors have read and approved the final manuscript.
